# Mobile Remote Monitoring of Intimate Partner Violence Among Pregnant Patients During the COVID-19 Shelter-In-Place Order: Quality Improvement Pilot Study

**DOI:** 10.2196/22790

**Published:** 2021-02-19

**Authors:** Tamar Krishnamurti, Alexander L Davis, Beth Quinn, Anabel F Castillo, Kelly L Martin, Hyagriv N Simhan

**Affiliations:** 1 Department of General Internal Medicine University of Pittsburgh School of Medicine Pittsburgh, PA United States; 2 Department of Engineering and Public Policy Carnegie Mellon University Pittsburgh, PA United States; 3 UPMC Magee-Womens Hospital Pittsburgh, PA United States; 4 Naima Health LLC Pittsburgh, PA United States; 5 Department of Obstetrics, Gynecology, and Reproductive Sciences University of Pittsburgh School of Medicine Pittsburgh, PA United States

**Keywords:** COVID-19, social isolation, sheltering-in-place, intimate partner violence, domestic violence, pregnancy, telemedicine, telehealth

## Abstract

**Background:**

Intimate partner violence (IPV) is one of the leading causes of pregnancy-related death. Prenatal health care providers can offer critical screening and support to pregnant people who experience IPV. During the COVID-19 shelter-in-place order, mobile apps may offer such people the opportunity to continue receiving screening and support services.

**Objective:**

We aimed to examine cases of IPV that were reported on a prenatal care app before and during the implementation of COVID-19 shelter-in-place mandates.

**Methods:**

The number of patients who underwent voluntary IPV screening and the incidence rate of IPV were determined by using a prenatal care app that was disseminated to patients from a single, large health care system. We compared the IPV screening frequencies and IPV incidence rates of patients who started using the app before the COVID-19 shelter-in-place order, to those of patients who started using the app during the shelter-in-place order.

**Results:**

We found 552 patients who started using the app within 60 days prior to the enforcement of the shelter-in-place order, and 407 patients who used the app at the start of shelter-in-place enforcement until the order was lifted. The incidence rates of voluntary IPV screening for new app users during the two time periods were similar (before sheltering in place: 252/552, 46%; during sheltering in place: 163/407, 40%). The overall use of the IPV screening tool increased during the shelter-in-place order. A slight, nonsignificant increase in the incidence of physical, sexual, and psychological violence during the shelter-in-place order was found across all app users (*P*=.56). Notably, none of the patients who screened positively for IPV had mentions of IPV in their medical charts.

**Conclusions:**

App-based screening for IPV is feasible during times when in-person access to health care providers is limited. Our results suggest that the incidence of IPV slightly increased during the shelter-in-place order. App-based screening may also address the needs of those who are unwilling or unable to share their IPV experiences with their health care provider.

## Introduction

Intimate partner violence (IPV) is one of the leading causes of pregnancy-related death [1-3]. In a study of pregnant women who were enrolled in the Nurse Family Partnerships program, 4.7% of participants reported that they experienced IPV during the first 36 weeks of their pregnancy [4]. The documented prevalence of IPV among pregnant women in the United States ranges from <1% to almost 20%, depending on the type of violence and the source of the estimate [5,6]. IPV prevalence estimates using hospital-based samples tend to be higher than population-based studies. This may be due to the higher number of documentation opportunities or the type of screening. Under shelter-in-place mandates, people who experience violence and abuse face restricted access to protective networks, such as health care professionals. Although increases in the number of IPV-related hotline calls have been reported in the media [7,8] and documented in scientific literature [9,10], there is still limited data on the increased number of IPV cases that have been reported to health care systems during the COVID-19 pandemic. Mobile apps that address IPV have been successfully designed [11]. Therefore, such apps may be effective sources of screening and support during the pandemic [12]. During enforced isolation, social networks are restricted. As such, mobile apps may also support the dissemination of IPV risk information to the public. In this study, we examined IPV cases that were reported through a prenatal care app that was prescribed to patients from a large academic health care system. We specifically examined cases from a single US county that were reported 2 months before and after the enforcement of shelter-in-place mandates in 2020.

## Methods

### Recruitment

In late September 2019, a large academic health system started prescribing the MyHealthyPregnancy app (Apple v.1.4.7 and Android v.1.8) [13] to pregnant patients during their first prenatal appointment. Prior to the COVID-19 pandemic, this app was poised to launch across the entire health care system as a quality improvement initiative. The MyHealthyPregnancy app uses machine learning algorithms to analyze patient-entered data, model each patient’s likelihood of experiencing adverse pregnancy events (eg, hypertension and premature delivery), and assess patients’ psychosocial risks. The app also offers relevant resources (eg, local women’s shelters) to users and notifies their providers about specific risk information in real time (ie, risk information that patients have shared on the app). Furthermore, all app users are notified that their care provider may not see or respond to all risk notifications. The app provides information on immediate actions that users can take to minimize their risks (eg, calling 911, calling their prenatal care provider, or watchfully waiting), based on the seriousness of the identified risks.

All app content was developed in conjunction with a clinical education team that was employed by the health care system. The same team reviewed all app content. The MyHealthyPregnancy app is a product of Naima Health LLC (limited liability company).

The internal protocol for prescribing the MyHealthyPregnancy app involved sending an invitation link to patients’ phones and prompting patients to use the app as part of their routine prenatal care. The link allowed users to access a unique code for downloading the app from the Android or Apple app store. By virtue of owning a smartphone, downloading the app, and completing the onboarding process, all users were considered smartphone and internet literate. An individual could not download the app and undergo the onboarding process unless they received a text message–based prescription for the app from their health care provider’s office.

The app users included in this study were pregnant residents of Allegheny County, Pennsylvania who were prescribed the MyHealthyPregnancy app during an in-person visit. All app users were offered an opportunity to undergo IPV screening through the app (ie, users received in-app messages that stated “Are you concerned about your safety? Take the pregnancy safety quiz”). Screening was not mandatory, and the screening tool could be accessed from a specific part of the app. We examined data that were collected from January 23 to May 15, 2020, to analyze app-based IPV disclosure before and during the enforcement of local shelter-in-place mandates (ie, approximately 2 months before and after mandate enforcement).

App users consented to the sharing of identifiable data for research purposes and the publication of anonymized aggregate data. Patients did not receive any financial compensation for app use, which was considered a part of routine prenatal care. All analyses were approved by the UPMC health system’s quality index review board.

### Safety Protocols

App access was protected by a password that was set by the user. The app included a password reset function that required users to input their user ID to receive a personalized text message that provided instructions on how to complete the process. A technical troubleshooting hotline was also available at all times. The IPV screening tool and screening-related notifications used language that was not specific to partner relationships or safety. These notifications were designed to be simple to dismiss, in case there were times when users felt uncomfortable with answering the screening questions. Furthermore, IPV screening data could not be stored or accessed in the app after the screening process was completed, thereby minimizing app users’ risk of privacy violations. An icon, which was displayed next to questions about IPV, informed users that positive IPV screening results would be sent to their health care provider. All positive IPV screening results were routinely sent to a UPMC clinical support team.

### Measures

The app onboarding process involved answering questions about clinical history (eg, prior preterm births and nulliparity) and demographics. Voluntary, validated screening measures were offered to users after they completed the onboarding process. We used two questions from the Centers for Disease Control and Prevention Behavioral Risk Factor Surveillance System as measures of physical violence and forced sexual acts. We also used 10 questions from the Women’s Experience with Battering scale to quantify psychological abuse [14,15]. Once shelter-in-place mandates were enforced, MyHealthyPregnancy app users were sent a text message that prompted them to use the app to share information about their COVID-19–related protective actions (eg, social distancing) with their care provider. All app users were also offered a COVID-19 triaging tool for assessing their symptoms.

IPV screening is part of the health care system’s standard prenatal screening battery. We reviewed the medical charts of patients who screened positively for IPV, to determine whether positive IPV screening tests were documented and to identify all in-person interactions that took place within the system (ie, emergency room, routine prenatal care, specialist care, and physical therapy appointment interactions) during the course of patients’ pregnancies.

### Statistical Analysis

We compared the IPV screening frequencies and IPV incidence rates of patients who started using the app before the shelter-in-place order, to those of patients who started using the app during the shelter-in-place order, by conducting a two-sample Chi-square test of proportions. IPV incidence rates were then queried against patients’ medical charts, to determine whether IPV was documented by a health care provider. Analyses were conducted with R version 3.6.0 (R Foundation for Statistical Computing).

## Results

In total, 552 users completed the MyHealthyPregnancy app onboarding process within 60 days prior to the enforcement of shelter-in-place orders (ie, January 23 to March 22, 2020), whereas 407 users completed the onboarding process between the start and end of shelter-in-place enforcement (ie, March 23 to May 15, 2020). Of the 284 respondents who answered all the questions about COVID-19 protective actions, approximately 281 (99%) reported that they adhered to shelter-in-place measures. Frequently performed protective actions included washing hands with soap and water multiple times per day; using hand sanitizer multiple times per day; wearing a face mask; and avoiding public spaces, gatherings, or crowds (eg, not socializing with people who had a high risk of SARS-CoV-2 infection or not frequenting restaurants). Table 1 shows the demographics of patients who completed the onboarding process before and during the shelter-in-place order.

**Table 1:** MyHealthyPregnancy app user demographics. The demographic distribution of the sample reflects the demographic distribution of the health care system’s pregnant population.

Variables		Completed onboarding before the shelter-in-place order (N=552), n (%)	Completed onboarding during the shelter-in-place order (N=407), n (%)
**Race/ethnicity**			
	Black/African American	62 (11)	40 (10)
	White	430 (78)	330 (81)
	Hispanic/Latinx	13 (2)	3 (1)
	Other	45 (8)	33 (8)
	Missing data	2 (<1)	1 (<1)
**Education status**			
	No high school or General Education Development diploma	24 (4)	13 (3)
	High school or General Education Development diploma	162 (29)	125 (31)
	Associate degree	58 (11)	56 (14)
	Bachelor's degree	155 (28)	93 (23)
	Postgraduate	150 (27)	117 (29)
	Missing data	3 (1)	3 (1)
**Relationship Status**			
	Single	20 (4)	21 (5)
	Ongoing relationship	159 (29)	115 (28)
	Married	369 (67)	269 (66)
	Divorced/separated	4 (1)	1 (<1)
	Missing data	0 (0)	1 (<1)

The number of patients who used the in-app IPV risk assessment tool did not differ significantly between patients who completed the onboarding process before the shelter-in-place order (252/552, 46%), and those who completed the onboarding process during the shelter-in-place order (163/407, 40%; two-sample Chi-square test of proportions: 95% CI −12% to −0.01%; *P*=.10). However, the app use rate of patients who completed the onboarding process before the shelter-in-place order was slightly lower than that of patients who completed the onboarding process during the shelter-in-place order. Moreover, the use of the in-app IPV risk assessment across all app users increased from 67% (368/552) to 85% (347/407) during the shelter-in-place order (95% CI 17%-28%; *P*<.001).

Patients who completed the onboarding process during the shelter-in-place order reported that they experienced similar levels of physical violence, sexual violence, and psychological abuse before and during the shelter-in-place order (Table 2). However, after considering all patients who had access to the app during the two time periods (Table 2), we observed a slight, but nonsignificant increase in the incidence of all forms of violence (*P*=.56). Notably, none of the physically at-risk patients (ie, those identified by the app) had any mentions of IPV in their medical chart. However, 24% (4/17) of physically at-risk patients received emergency room care, and 29% (5/17) underwent physical therapy or a prenatal consultation for nonspecific pain or injury.

**Table 2:** Reports of intimate partner violence and the results of intimate partner violence screening.

Type of intimate partner violence	Before shelter-in-place order			During shelter-in-place order		
	New unique screens vs new unique patients, n/N (%)	New unique positive reports vs new unique patients, n/N (%)	All positive reports vs all reports, n/N (%)	New unique screens vs new unique patients, n/N (%)	New unique positive reports vs new unique patients, n/N (%)	All positive reports vs all reports, n/N (%)
Physical violence^a^	252/552 (46)	2/552 (0.4)	4/461 (0.87)	163/407 (40)	2/407 (0.5)	6/443 (1.4)
Sexual violence^b^	252/552 (46)	2/552 (0.4)	3/461 (0.65)	163/407 (40)	1/407 (0.2)	4/443 (0.9)
Psychological abuse^c^	252/552 (46)	6/552 (1)	6/461 (1.3)	163/407 (40)	3/407 (0.7)	6/442 (1.4)

^a^A 1-item measure (ie, “Has an intimate partner or ex-partner, hit, slapped, kicked, choked, or otherwise physically hurt you in the past month?”).

^b^A 1-item measure (ie, “Has an intimate partner or ex-partner hit, coerced or forced you into sexual activity against your will in the past month?”).

^c^A 10-item measure based on the Women’s Experience with Battering scale (eg, “I try not to rock the boat because I am afraid of what my partner might do”).

## Discussion

### Principal Results

In this study, we analyzed a stable, prenatal care app–based IPV self-screening tool that was used during shelter-in-place conditions. During the shelter-in-place order, there was a slight, but nonsignificant increase in the incidence of all forms of IPV (*P*=.56). This finding suggests that during times of social isolation (ie, the COVID-19 pandemic), people continue to use technology to disclose their concerns about an increased risk of IPV to care providers [16,17]. However, care providers should take into account the unique needs of pregnant women, to provide them with opportunities for mitigating pregnancy-related risks. It should be noted that even though we observed a nonsignificant, increasing trend in IPV incidence (*P*=.56), new app users’ engagement with the IPV risk assessment tool was stable during both time periods, while overall use went up. One explanation for this is that during the COVID-19 pandemic, people have limited opportunities to seek help outside of home. Therefore, people may be delaying seeking help until abuse starts to escalate.

Given that the people who screened positively for IPV by using an app-based tool were not documented in routine, in-person screening, we believe that the MyHealthyPregnancy app may address the needs of patients who are unable or prefer not to disclose their IPV experiences directly to their care provider. App-based screening may serve as a complementary form of care for those with limited access to health care providers or other social networks. Such technologies may offer patients an additional method for communicating with care providers during times of limited mobility and care access.

### Limitations

IPV is serious, and even though it occurs frequently, IPV is rarely reported, even during routine, in-person screening with trusted health care providers. Although our results suggest that there was an increase in the incidence of IPV during the shelter-in-place order, our sample size was not large enough to detect statistically significant differences. This is most likely due to the low rates of disclosure among pregnant women who experience IPV. Although we hope that app-based screening will provide patients with an additional layer of support (ie, by making screening and appropriate resources available at any time), the decision to undergo screening and seek resources is voluntary, and many people may not feel safe or ready to disclose their IPV-related experiences, especially if they are sheltering in place with an abusive partner.

Another limitation of this study is that we were unable to determine whether app-users who screened positively for IPV actually used the resources that were offered through the app. However, we are currently working on a warm handoff system for providing resources to patients who screen positively for IPV and wish to connect with such resources. Although the app users in our study reflect the general population of patients in our health system, it is possible that people who choose not to use the MyHealthyPregnancy app are more likely to experience IPV. This would result in less effective screening. However, we cannot discern this based on our data. It is also possible that app users who experienced IPV, but chose not to disclose it, reviewed or accessed the universally available support resources that the MyHealthyPregnancy app offers. We hope that this was the case.

### Comparison With Prior Work

Regardless of social distancing mandates, the fact that none of the app-detected IPV cases were documented during emergency room visits or other clinical visits highlights a gap in prenatal care [18] that can be filled by implementing app-based technology methods. After shelter-in-place restrictions are lifted, the transition to telemedicine protocols may result in more instances of remote routine screening. Although our results only suggest that pregnant women are more willing to disclose IPV experiences through an app than they are during an in-person encounter, offering app-based IPV risk screening and appropriate resources is one way to address the needs of pregnant people who experience violence during and beyond times of social isolation. Moreover, app-based screening may offer an additional layer of support to patients who would benefit from universally available resources, but do not receive them, even after they present with physical signs of abuse during in-person care visits. This method of screening also allows care providers to anonymously identify the common signs of abuse and violence that patients may present with when seeking care [19] but are missed during in-person visits.

In this study, the number of patients who screened positively for IPV before and during the shelter-in-place period reflects the lower end of national estimates for IPV incidence rates. Therefore, it is unlikely that we identified all instances of IPV among app users (ie, it is possible is that patients who experience IPV do not frequently use smartphones or apps). As such, it is important to take into account that app-based screening alone does not necessarily identify all instances of IPV, especially if an abusive partner controls smartphone use. Our results also suggest that app-based screening may capture a different set of patients who are at risk of IPV, which means that an app-based approach can be used to complement in-person assessments. An app-based approach also provides patients (ie, those who feel that they may be at risk of IPV) with the freedom to engage with screening tools when they feel safe and comfortable.

## Appendix

Multimedia Appendix 1 CONSORT-eHEALTH checklist (V 1.6.1).

## Abbreviations

IPV: intimate partner violence
LLC: limited liability company
